# Ischemic Heart Disease Selectively Modifies the Right Atrial Appendage Transcriptome

**DOI:** 10.3389/fcvm.2021.728198

**Published:** 2021-12-02

**Authors:** Severi Mulari, Arda Eskin, Milla Lampinen, Annu Nummi, Tuomo Nieminen, Kari Teittinen, Teija Ojala, Matti Kankainen, Antti Vento, Jari Laurikka, Markku Kupari, Ari Harjula, Nurcan Tuncbag, Esko Kankuri

**Affiliations:** ^1^Department of Pharmacology, Faculty of Medicine, University of Helsinki, Helsinki, Finland; ^2^Heart and Lung Center, University of Helsinki and Helsinki University Hospital, Helsinki, Finland; ^3^Department of Health Informatics, Graduate School of Informatics, Middle East Technical University (METU), Ankara, Turkey; ^4^Department of Oral and Maxillofacial Diseases, Faculty of Medicine, University of Helsinki, Helsinki, Finland; ^5^Research Programs Unit, University of Helsinki, Helsinki, Finland; ^6^Department of Cardiothoracic Surgery, Heart Center, Tampere University Hospital, Tampere, Finland; ^7^Faculty of Medicine and Health Technology, Tampere University, Tampere, Finland; ^8^Department of Chemical and Biological Engineering, College of Engineering, Koc University, Istanbul, Turkey; ^9^School of Medicine, Koc University, Istanbul, Turkey

**Keywords:** biomarkers, ischemia, chronic ischemic heart disease, cardiovascular surgery, atrial appendage, differentially expressed genes

## Abstract

**Background:** Although many pathological changes have been associated with ischemic heart disease (IHD), molecular-level alterations specific to the ischemic myocardium and their potential to reflect disease severity or therapeutic outcome remain unclear. Currently, diagnosis occurs relatively late and evaluating disease severity is largely based on clinical symptoms, various imaging modalities, or the determination of risk factors. This study aims to identify IHD-associated signature RNAs from the atrial myocardium and evaluate their ability to reflect disease severity or cardiac surgery outcomes.

**Methods and Results:** We collected right atrial appendage (RAA) biopsies from 40 patients with invasive coronary angiography (ICA)-positive IHD undergoing coronary artery bypass surgery and from 8 patients ICA-negative for IHD (non-IHD) undergoing valvular surgery. Following RNA sequencing, RAA transcriptomes were analyzed against 429 donors from the GTEx project without cardiac disease. The IHD transcriptome was characterized by repressed RNA expression in pathways for cell–cell contacts and mitochondrial dysfunction. Increased expressions of the *CSRNP3, FUT10, SHD, NAV2-AS4*, and hsa-mir-181 genes resulted in significance with the complexity of coronary artery obstructions or correlated with a functional cardiac benefit from bypass surgery.

**Conclusions:** Our results provide an atrial myocardium-focused insight into IHD signature RNAs. The specific gene expression changes characterized here, pave the way for future disease mechanism-based identification of biomarkers for early detection and treatment of IHD.

## Introduction

The early diagnosis of ischemic heart disease (IHD) could facilitate the design of therapeutic strategies and, thus, improve patient prognosis (1). To this end, IHD biomarkers which specifically and sensitively correlate with disease severity are required (2). Currently, initial assessment of IHD is largely based on the patient's clinical symptoms, distinct electrocardiographic (ECG) alterations, and risk scoring. Diagnosis depends on cardiac stress testing and radiographic evaluations of coronary vessel obstructions or myocardial perfusion (3, 4). These are supplemented with laboratory investigations (5). These surrogate markers, however, do not directly reflect the myocardial structure, metabolism, or inflammation (6). We propose that the right atrial appendage (RAA), safely sampled during heart surgery, can provide direct insight into the pathophysiological myocardial changes manifesting in IHD. For the further discovery of IHD biomarkers, it is crucial to understand these changes and to utilize them for laying the rational pathology-based groundwork in molecular detail. Toward this, we collected 48 RAA biopsies from patients with or without IHD, and compared their RNA-sequenced (RNAseq) transcriptomes to those from RAA biopsies from 429 donors without heart disease available from the Genotype Tissue Expression (GTEx) project. In doing so, we establish RAA as a feasible source for the functional and metabolic characterization of cardiac ischemic pathologies, and show that specific RAA gene expressions correlate with IHD complexity and the functional benefit of cardiac surgery.

## Materials and Methods

### Ethics

This study was approved by the Operative Ethics Committee of the Hospital District of Helsinki and Uusimaa (DNro 286/13/03/02/2012). The study was registered to the ISRCTN registry (ISRCTN15411573). Before surgery, all patients provided their written informed consent and received treatment adhering to evidence-based clinical guidelines as routinely adopted by healthcare centers.

### Patients and Samples

For this study, we recruited a total of 54 patients scheduled to undergo elective cardiac surgery between 2014 and 2015 at the Heart and Lung Center at Helsinki University Central Hospital (Helsinki, Finland) or Tampere Heart Hospital, Tampere University Hospital (Tampere, Finland). We assigned 48 patients to the RAA RNA sequencing arm and 6 patients to the RNA sequencing data validation arm. Within the RNA sequencing arm, 35 patients (35/48, 73%) underwent CABG surgery, 8 patients (8/48, 17%) underwent aortic valve surgery, and combined CABG and aortic valve surgery was performed on 5 patients (5/48, 10%). According to invasive coronary angiography (ICA), patients were categorized into IHD or non-IHD groups. The IHD group (*n* = 40) included patients with ICA occlusions, and contained patients who underwent CABG or combined surgery. The non-IHD group (*n* = 8) included patients with no ICA occlusions, and containing patients who underwent isolated valvular surgery. All 6 patients in the validation arm underwent CABG surgery and had ICA occlusions. We used RAA RNAseq transcriptomes from donors (*n* = 429) without heart disease from the GTEx biobank as the controls. Causes of deaths among GTEx control patients were classified according to the four-point Hardy Scale in order to exclude cases with a cardiac background. Detailed patient demographic characteristics are provided in Supplementary Table 1.

During cardiac surgery, a biopsy sample of the RAA was collected from each patient. Tissue samples were collected at the cannulation site, the insertion site of the venous line for the heart-lung machine. Dissected biopsies were briefly washed in ice-cold sterile saline, cut into 0.5 cm-thick tissue pieces, and immersed into 3 ml of RNAlater (Sigma Aldrich, Merck KGaA, Darmstadt, Germany) to inactivate RNAses and stabilize RNA. Samples were left to stabilize at +4°C for 1 day and were thereafter stored at −70°C or below until processing. The RAA tissues of the validation arm patients were collected into formalin for fixation at +4°C for 14 days, followed by storage in 70% ethanol until processing for paraffin embedding.

Total RNA was isolated from RAA biopsies via initial ice-cold tissue homogenization using a Precellys 24 tissue homogenizer equipped with the Cryolys cooling option (Bertin Technologies SAS, Montigny-le-Bretonneux, France) followed by RNA isolation using combined QIAzol/chloroform extraction and Qiagen RNeasy mini spin column purification according to the manufacturer's instructions (Qiagen, Venlo, Netherlands). The sample RNA integrity number (RIN; mean 7.95 ± 0.55; range 7.1–9.0) and total quantity (mean 3069.55 ± 1141.08 ng; range 867.8–6855.0 ng) were evaluated in the Functional Genomics Unit (FuGU, University of Helsinki, Helsinki, Finland). Isolated total RNA was stored at −70°C or below until sequencing. RNA sequencing was performed by the BGI Group (Copenhagen, Denmark). Ribosomal RNA was removed using the Ribo-Zero^TM^ Magnetic kit (Illumina Inc., San Diego, CA), and the samples were sequenced using the BGI LncRNA-seq protocol with Illumina HiSeq 4000 technology (100PE, BGI Group).

### Collection of Patient Data

Cardiovascular disease and treatment-related clinical data were collected from electronic healthcare records. Missing values were retrieved and reanalyzed from the stored raw sequences, including those from echocardiography, electrocardiography, and angiography. Drug treatment data from the hospital databases were meticulously recorded from 6-month pre- and post-operative periods. To enable drug treatment comparisons, we used the InnoLIMS Medical software (Innovatics Oy, Helsinki, Finland) to categorize and standardize each treatment according to its classification in the Anatomical Therapeutic Chemical (ATC) classification system and by converting each daily dosing regimen to the corresponding defined daily dose (DDD, Finnish Medicines Agency, FIMEA, Helsinki, Finland and the World Health Organization Collaborating Center for Drug Statistics Methodology, Oslo, Norway). To capture patients' stable medication regimens unrelated to hospitalization, treatments were recorded at 3 weeks preoperatively and 6 months post-operatively.

### Data Analysis of RNA-Sequenced Data

Fastq files were trimmed using Trimmomatic v0.32 (7) applying the following parameters: ILLUMINACLIP: < fasta with adapters>:2:30:10 LEADING:3 TRAILING:3 SLIDINGWINDOW:4:15 MINLEN:36. Trimmed reads were mapped to the reference genome (EnsEMBL gene collection v82) (8) using STAR v2.3.0 (9) with the following parameters: –genomeLoad NoSharedMemory –readFilesCommand zcat –outSAMattributes All –outSAMunmapped Within. For read quantification, we used featureCounts (10) with the following parameters: “-F” “GTF” “-t” “exon” “-g” “gene_id” “-O” “-T” “16” “-a.” Gene counts from 429 healthy tissue samples were obtained from the GTEx analysis release V8. For subsequent analyses, only genes with an average logCPM >1 were considered. We used R bioconductor's package edgeR (11) to normalize the read counts and test for differentially expressed genes. We performed upper quartile normalization on the read counts, which were fitted to a quasi-likelihood negative binomial generalized log-linear model. In addition, we performed the quasi-likelihood *F*-test using the glmQLFit and glmQLFtest functions. Finally, differentially expressed genes were selected applying |logFC| >2 and FDR <0.05.

The RNA sequencing data is available from the Gene Expression Omnibus database repository under the identification GSE173594, https://www.ncbi.nlm.nih.gov/geo/query/acc.cgi?acc=GSE173594.

### Functional Enrichment Analysis

We carried out functional enrichment analyses using IPA software (Qiagen). Enriched pathways were selected using FDR <0.05 for differentially expressed genes from ischemic, non-ischemic, and healthy tissue samples. For the miRNA targets, we selected enriched pathways applying *p* < 0.05. The functional annotation of genes significantly downregulated or upregulated across surgery benefit groups was extracted separately from the diseases, and the functions tab for IPA, enriched diseases, and functions were selected by excluding cancer-related results and applying FDR <0.05.

### mIRNA Targets

miRNA targets were identified using Targetscan 7.2 (12). Targetscan calculates context++ score for the complementary site of miRNA by summing contributions from 14 features. Some of the features are site type, 3′ UTR length, and the target site abundance. The total context++ score is calculated for a representative transcript by summing the context++ scores for the sites to the representative miRNA. Many of the targets for a specific miRNA are false positives in the Targetscan, requiring a cutoff. Thus, targets were extracted using a cutoff total context++ score <-0.8. We found that with a less stringent cutoff, the number of targets increased greatly. This specific cutoff was selected to extract high confidence targets.

### Quantitative and Qualitative Patient Categorizations

In order to quantitatively evaluate the complexity of coronary artery obstructions, preoperative SYNTAX scores were calculated for all patients. Patients were classified into three groups based on their SYNTAX score values using cut-offs from the US/European guidelines [low ≤ 22 (*n* = 34), intermediate 23–32 (*n* = 10), and high score >33 (*n* = 4)] (13, 14). Moreover, information on occlusion of proximal RCA was also collected in order to perform subanalysis correlating proximal RCA occlusion and gene expression of RAA. Major adverse cardiovascular and cerebrovascular events (MACCE) relied on a four-point MACCE score: death due to a cardiovascular reason, non-fatal stroke, non-fatal myocardial infarction, and hospitalization due to heart failure (HF). MACCE and mortality rates were recorded from a 5-year postoperative follow-up.

Surgery benefit was assessed by calculating the preoperative and post-operative ejection fraction (EF). Preoperative EF was evaluated within 1 month before surgery, and post-operative EF was determined during a 3-month control visit. An EF change was calculated (postoperative EF – preoperative EF), and patients were divided into two groups according to the result: (a) good benefit group (postoperative EF >10% better than preoperative EF) and (b) poor benefit group (postoperative EF reduced >10% compared to preoperative EF).

### Validation

The six formalin-fixed AA samples for validation were paraffin-embedded at the Tissue Preparation and Histochemistry Unit (Faculty of Medicine, University of Helsinki, Finland). Immunohistochemistry for FUT10 and CSRNP3 was performed using standardized protocols at BioSiteHisto Ltd (Tampere, Finland) and the primary antibodies validated in the Human Protein Atlas project (www.proteinatlas.org, rabbit anti-human CSRNP3 HPA017905 used here at dilution 1:75, and rabbit anti-human FUT10 HPA053970 used here at dilution 1:50; both from Sigma Aldrich, Merck KGaA, Darmstadt, Germany). Staining was cross-validated against in-house human tissue control sections. The immunostained RAA tissue sections were scanned with the 3d-HISTECH Pannoramic MIDI II imaging system using a x20 objective at 0.23 × 0.23 μm/pixel resolution (3DHISTECH Ltd., Budapest, Hungary). For analysis, serial non-overlapping images at × 20 magnification covering each section were captured with the Pannoramic Viewer software (3DHISTEC Ltd.). The analysis of the captured images was carried out using the FiJI ImageJ software (15). The image analysis macros are available upon request from the corresponding author. Briefly, for each image, after background subtraction, the color deconvolution algorithm to hematoxylin (H) and diaminobenzidine (DAB) channels was utilized. The DAB channel image was thresholded to the stain using the automated default method based on the IsoData algorithm, and the stain intensity was measured. For nuclear counting, hematoxylin-positive nuclei (representing the total amount of nuclei in the image) were counted from the H channel using the particle-counting algorithm and compared with the thresholded positively immunostained nuclei (stained nuclei) counted from the DAB channel. The results of the densitometric image analysis of the serial images for each sample were first averaged, and these single values were then used for the further combined analysis of results.

### Statistical Analyses

Differentially expressed genes between phenotypic ischemic and non-ischemic patient groups were identified by performing permutation tests using gene TPMs (Transcripts per Million). Results from the permutation test were corrected using the Benjamin-Hochberg FDR correction (FDR <0.05). Correlation analyses relied on Pearson's correlation comparing blood test parameters and gene TPMs, for which we applied *p* < 0.05. Genes associated with the SYNTAX score groups were identified through a one-way ANOVA (*p* < 0.05) using the TPM values for genes that were differentially expressed compared to GTEx. For the clinical data, statistical differences in continuous variables were calculated using the Kruskal-Wallis test (three groups) and the Mann-Whitney *U*-test (two groups). Statistical differences in categorical variables were calculated using Fisher's exact test. We considered *p* < 0.01 to denote statistical significance, and all *p*-values were two-tailed. Clinical data were analyzed using SPSS (IBM's SPSS Statistics for Windows, version 25.0, IBM Corp., Armonk, NY).

## Results

**Figure 1:**
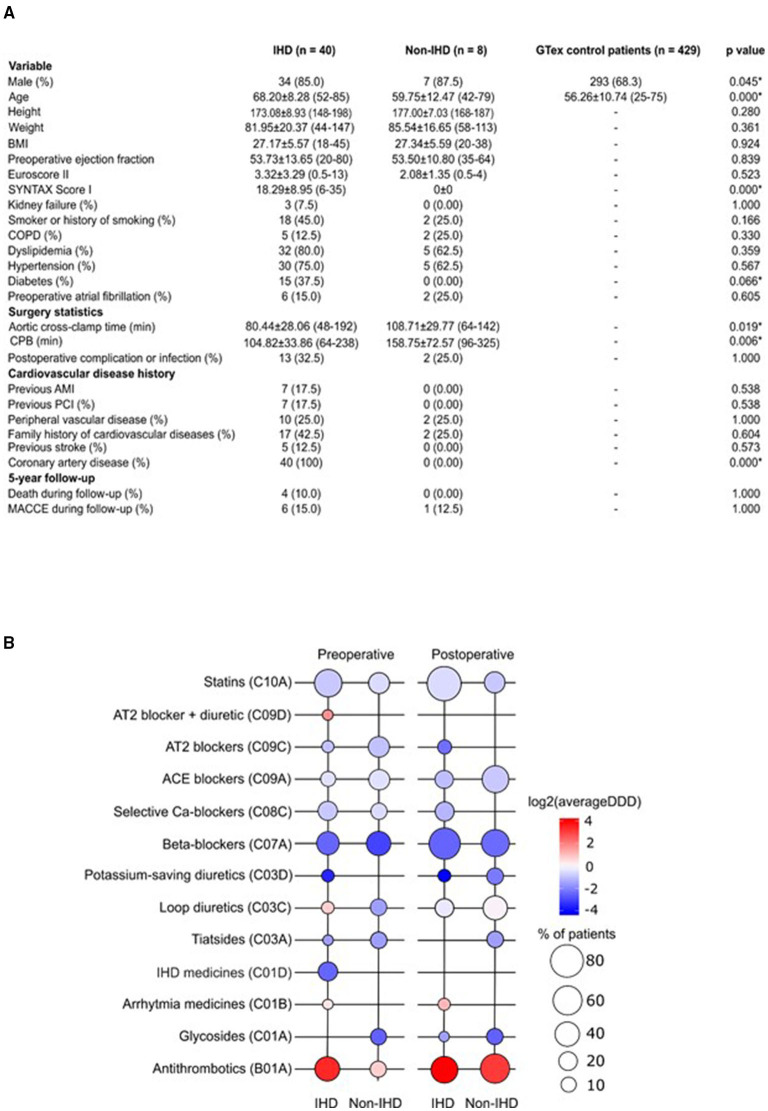
**(A)** Demographic characteristics of the study population. AMI, acute myocardial infarction; BMI, body mass index; COPD, chronic obstructive pulmonary disease; CPB, cardiopulmonary bypass; MACCE, major adverse cardiovascular and cerebrovascular event; PCI, percutaneous coronary intervention. **(B)** Pre- and post-operative medication use in the study groups. Color key representing the total use of medication (as log_2_ average DDD); bubble plot representing the percentage of patients on medication indicated in study groups. DDD, defined daily dose.

Patients in the GTEx and non-IHD groups were younger (mean age 56.26 years ± 10.74 and 59.75 ± 12.47, respectively) than IHD patients (mean age 68.20 ± 8.28) (Figure 1A). While the mean SYNTAX score was 0 in the non-IHD group, in the IHD group it reached 18.29 ± 8.95. In addition, diabetes occurred more frequently in the IHD group. Aortic cross-clamp times and cardiopulmonary bypass (CPB) times were relatively longer in the non-IHD group.

Preoperative medication in the IHD group reflected ischemic disease, particularly the use of organic nitrates; no organic nitrate use was recorded among non-IHD patients (Figure 1B). Post-operatively, the dyspnea- and chest pain symptom-alleviating effects of CABG surgery were reflected as an extinct use of organic nitrates in IHD patients. Drug treatments were overall more uniform following surgery. Postoperatively, statin treatment was more common among IHD patients (*p* = 0.006). We detected differences in postoperative complications or infections when comparing groups. In total, three IHD patients, but no non-IHD patients, were treated with antiarrhythmic medication during the preoperative and postoperative periods. Overall, ACE inhibitors and antithrombotics were used more during the post-operative period.

### DEGs Between IHD and Non-IHD Compared to GTEx Database

**Figure 2:**
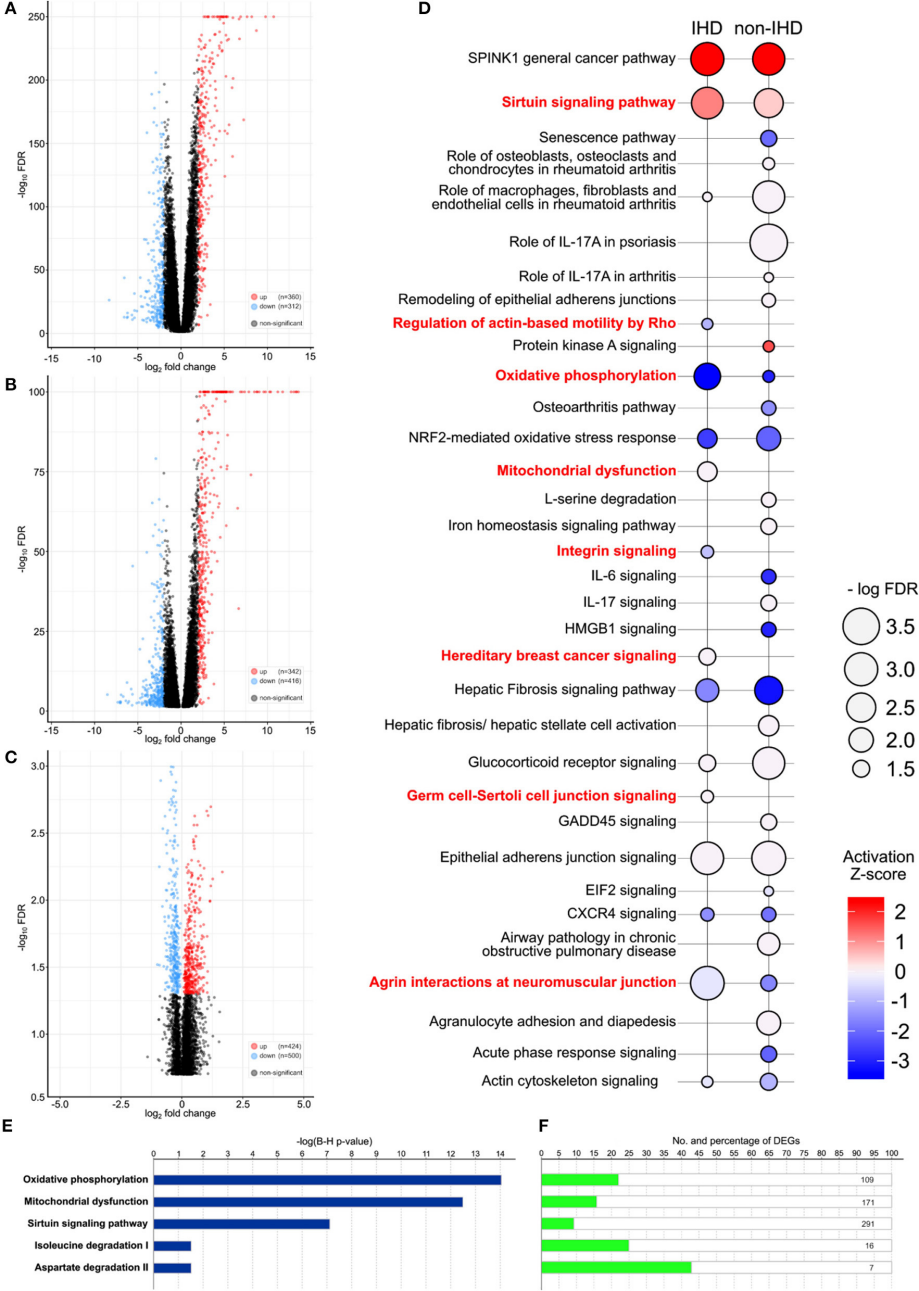
**(A–C)** Volcano plots providing an overview and the number of differentially expressed genes (DEGs). IHD vs. GTEx patients **(A)**, non-IHD vs. GTEx patients **(B)**, and IHD vs. non-IHD patients **(C)**. **(D)** DEG-related pathways between IHD and non-IHD vs. GTEx comparison. Major differences are highlighted in red, with the color key showing the activation Z-score. The size of a balloon represents the –log(FDR) value. **(E)** *P*-values for the top five enriched pathways in IHD vs. non-IHD group comparisons. **(F)** Number of DEGs in the top five enriched pathways IHD vs. non-IHD group comparisons.

A comparison of the gene expression profiles of the IHD group and GTEx controls yielded 672 differentially expressed genes (DEGs), 360 of which were upregulated and 312 downregulated (Figure 2A). Sirtuin signaling, epithelial adherens junction signaling, and serine peptidase inhibitor Kazal type 1 (SPINK1) were significantly upregulated in the IHD group. In contrast, agrin interactions and oxidative phosphorylation were significantly downregulated (Figure 2D). In the non-IHD vs. GTEx comparison, we identified 342 upregulated and 416 downregulated DEGs (Figure 2B). DEGs correlated with inflammatory and immunological processes (Figure 2D). The natriuretic peptide B (NPPB) coding gene was differentially expressed in both the IHD (logFC −2.65, *p* < 0.001) and non-IHD (logFC −4.08, *p* = 0.001) groups vs. the GTex group.

### Differences Between IHD and Non-IHD Groups

Comparison of IHD and non-IHD samples resulted in 424 significantly upregulated and 500 significantly downregulated genes (Figure 2C). DEGs were enriched in oxidative phosphorylation, mitochondrial dysfunction, sirtuin signaling, isoleucine degradation, and aspartate degradation (Figures 2E,F). The two latter pathways strengthen previous associations identified for branched-chain amino acids and aspartate as metabolic contributors to IHD (16, 17). As such, our results directly situate myocardial changes in these amino acid pathways as the culprit in IHD.

**Figure 3:**
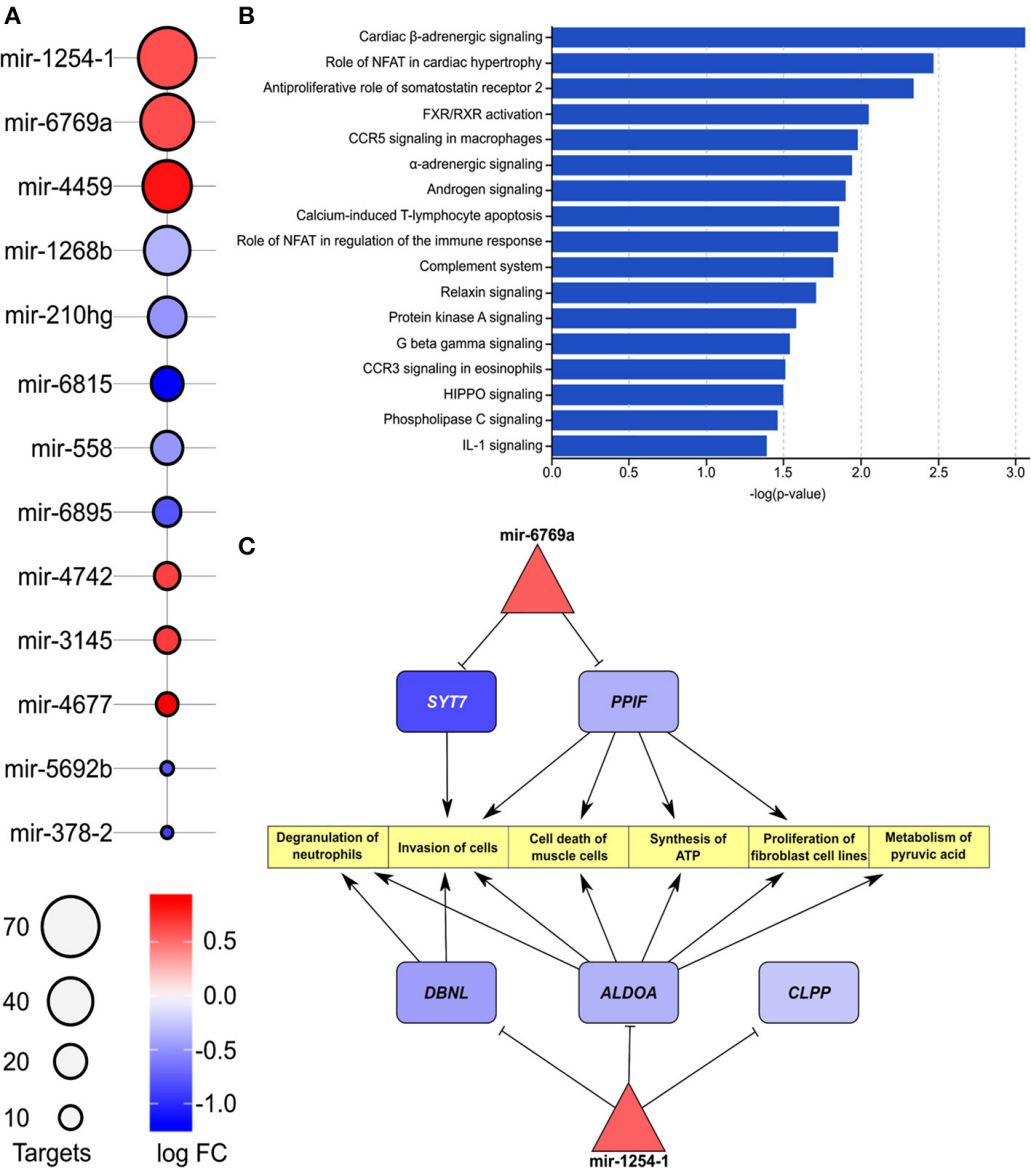
**(A)** List of enriched miRNAs in IHD vs. non-IHD patients. Color key, log FC; bubble plot, number of targets. **(B)** List of pathways related to enriched miRNAs. The bars express –log(*p*-value). **(C)** Differentially expressed downstream target genes and pathways related to hsa-mir-1254-1 and hsa-mir-6769a.

The list of differentially expressed miRNAs, significant downstream pathways, and functionally annotated targets of these miRNAs from comparison of IHD and non-IHD is shown in Figures 3A,B. We found a total of 13 differently expressed miRNAs between groups (Figure 3A). The target genes of these miRNAs are significantly enriched in cardiac β-adrenergic signaling, role of NFAT in cardiac hypertrophy, androgen signaling, and complement system pathways. A three-layer subnetwork of interactions between miRNA, their differentially expressed target genes, and their functions appears in Figure 3C. Most of the miRNA targets in this subnetwork are downregulated. These genes play a role in muscle cell death, metabolizing pyruvic acid, fibroblast cell line proliferation, cell invasion, synthesizing ATP, and degranulating neutrophils.

### Clinical Associations to Preoperative Condition

#### Ejection Fraction

**Figure 4:**
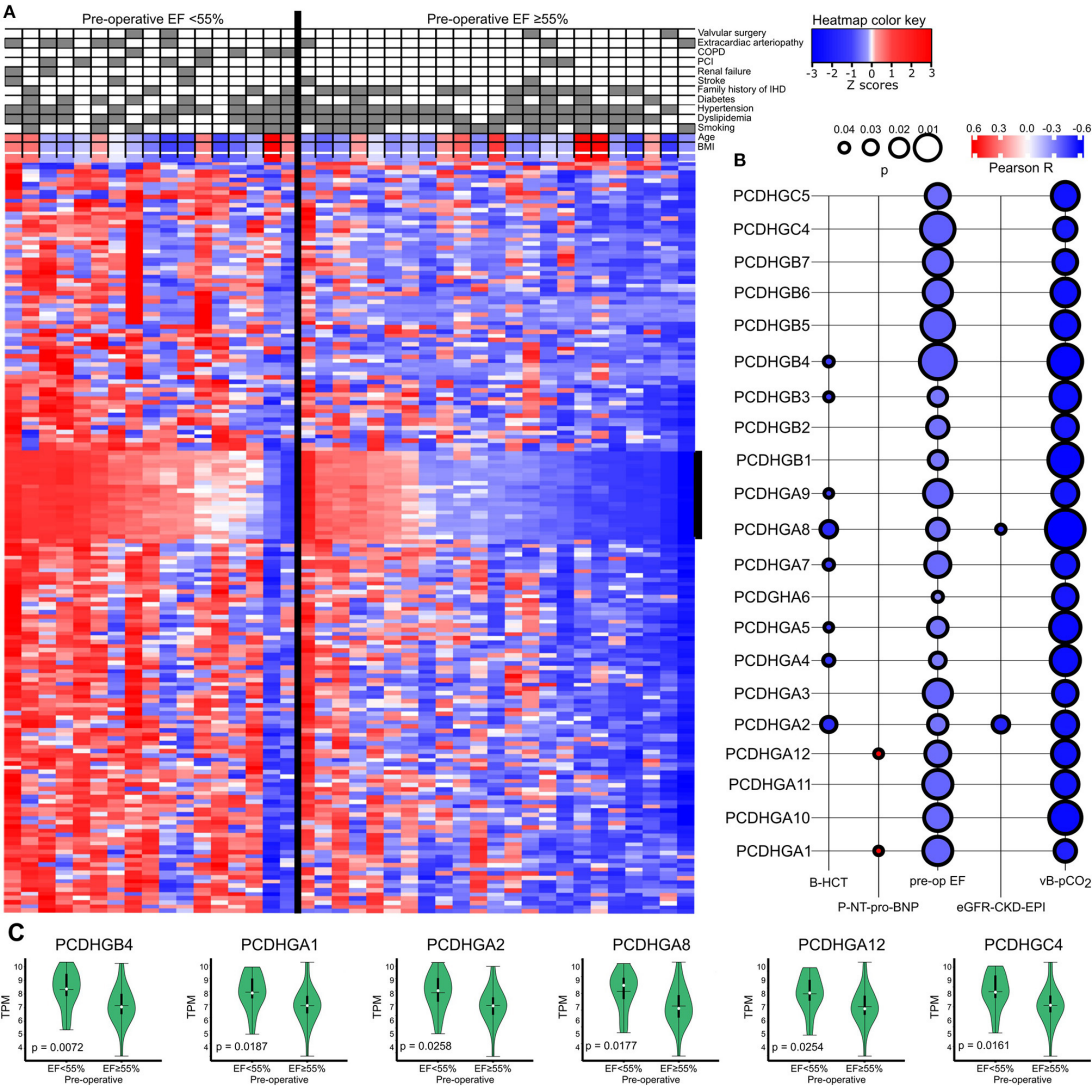
**(A)** Heatmap showing a general overview of DEGs comparing the categorized EF groups in IHD patients. The heatmap color key expresses the Z-scores. **(B)** Correlations between PCDHGs and preoperative EF and preoperative laboratory values. The size of the bubble indicates the *p*-value and the color key indicates the Pearson R. **(C)** Correlations between preoperative EF and PCDHGs. EF, ejection fraction; PCDHG, protocadherin.

We further divided IHD patients into two groups based on a high (≥55%) or low (<55%) preoperative EF value and clustered the genes accordingly (Figure 4A). Protocadherin gamma family members (PCDHGs) exhibited higher expression in the low preoperative EF group (EF <55%). The correlation between PCDHGs and preoperative laboratory tests appear in Figure 4B. *PCDHGA1* and *PCDHGA12* positively correlated with preoperative NT-proBNP levels (Pearson *R* = 0.6, *p* = 0.04), while all PCDHGs negatively correlated with preoperative EF values (Figure 4B). In addition, Figure 4C displays selected PCDHGs and their expressions based on the preoperative EF groups. Each selected PCDHG is highly expressed in patients in the low EF group compared to the high EF group. These results further strengthen the association of the *PCDHG* gene cluster with cardiac dysfunction (18).

#### SYNTAX Score and Laboratory Measurements

**Figure 5:**
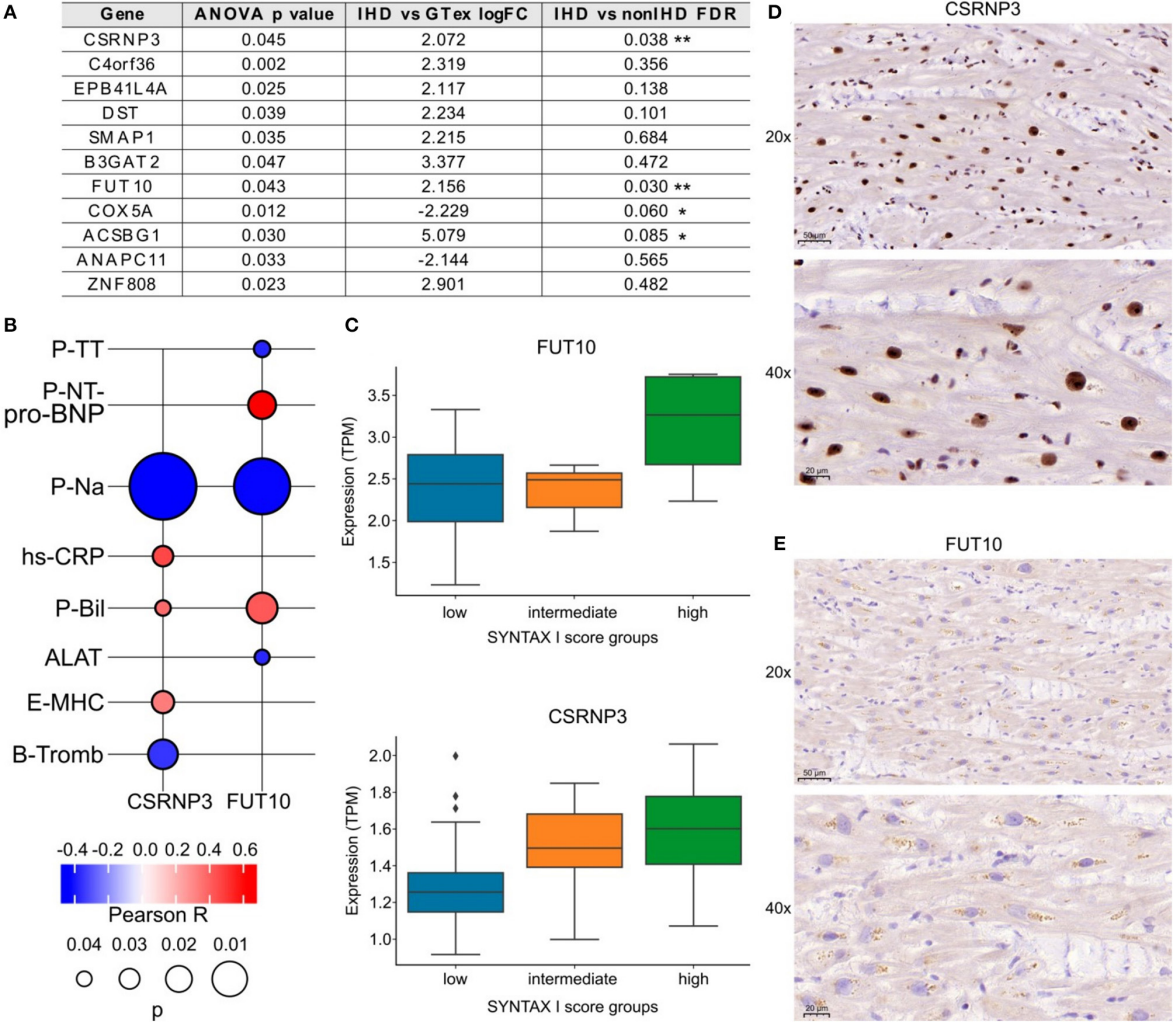
**(A)** List of genes associated with SYNTAX score I. **(B)** *FUT10* and *CSRNP3* correlations with preoperative laboratory tests. The color key illustrates the Pearson R and the size of the bubble indicates the *p*-value. **(C)** *FUT10* and *CSRNP3* expression in the SYNTAX score groups. **(D,E)** Representative *CSRNP3* and *FUT10* stainings from the validation group.

We categorized the patients into three groups according to low, intermediate, or high SYNTAX scores reflecting the level of coronary artery obstruction complexity. All IHD patients except one had obstructions also in the right coronary artery (RCA). Total of 30 patients had occlusion in the proximal RCA, 7 patients in the mid RCA and 2 in the distal RCA. We identified 11 DEGs across the groups (ANOVA test, Figure 5A). Among them, *CSRNP3* (Cysteine and Serine Rich Nuclear Protein 3) and *FUT10* (Fucosyltransferase 10) genes were common in DEGs in the IHD vs. non-IHD groups (ANOVA *p* = 0.045 and *p* = 0.043; FDR 0.036 and 0.030, respectively). Although the differential expression of *COX5A* (Cytochrome C Oxidase Subunit 5A) and *ACSBG1* (Acyl-CoA Synthetase Bubblegum Family Member 1) suggested IHD selectivity, with their expressions significantly correlating with the SYNTAX scores (ANOVA *p* = 0.012 and *p* = 0.030; FDR 0.060 and 0.085, respectively), this difference did not emerge in the DEG comparison between the IHD compared with non-IHD groups.

The associations between *FUT10* and *CSRNP3* and the preoperative laboratory values appear in Figure 5B. *FUT10* positively correlated with preoperative NT-proBNP levels (Pearson *R* = 0.6; *p* = 0.03), whereas *CSRNP3* positively correlated with the blood CRP levels (Pearson *R* = 0.4; *p* = 0.03). The expressions of *CSRNP3* and *FUT10* were higher in the high SYNTAX score category (Figure 5C), suggesting that lower *CSRNP3* and *FUT10* expression levels reflect milder or less complex coronary artery disease. Figures 5D,E provide representative immunohistochemistry images of the validation arm RAA samples with primary antibodies against the *FUT10* and *CSRNP3* proteins. The results from the immunohistochemistry analysis and box plots showing the percentage of area staining for *CSRNP3* and *FUT10* compared to the total sample area appear in Supplementary Figure 1. We identified no statistically significant differences although FUT10 protein expression presented with an increased tendency in the high SYNTAX score group. Further analysis of the *FUT10* transcripts revealed the highest expression for the *FUT10-201* transcript (median TPM >2.5) in our study population (Supplementary Figure 2) suggesting differential regulation of alternate transcription. Results from RCA-occlusion subanalysis are presented in Supplementary Figure 3.

### Molecular Associations With Surgery Benefit

**Figure 6:**
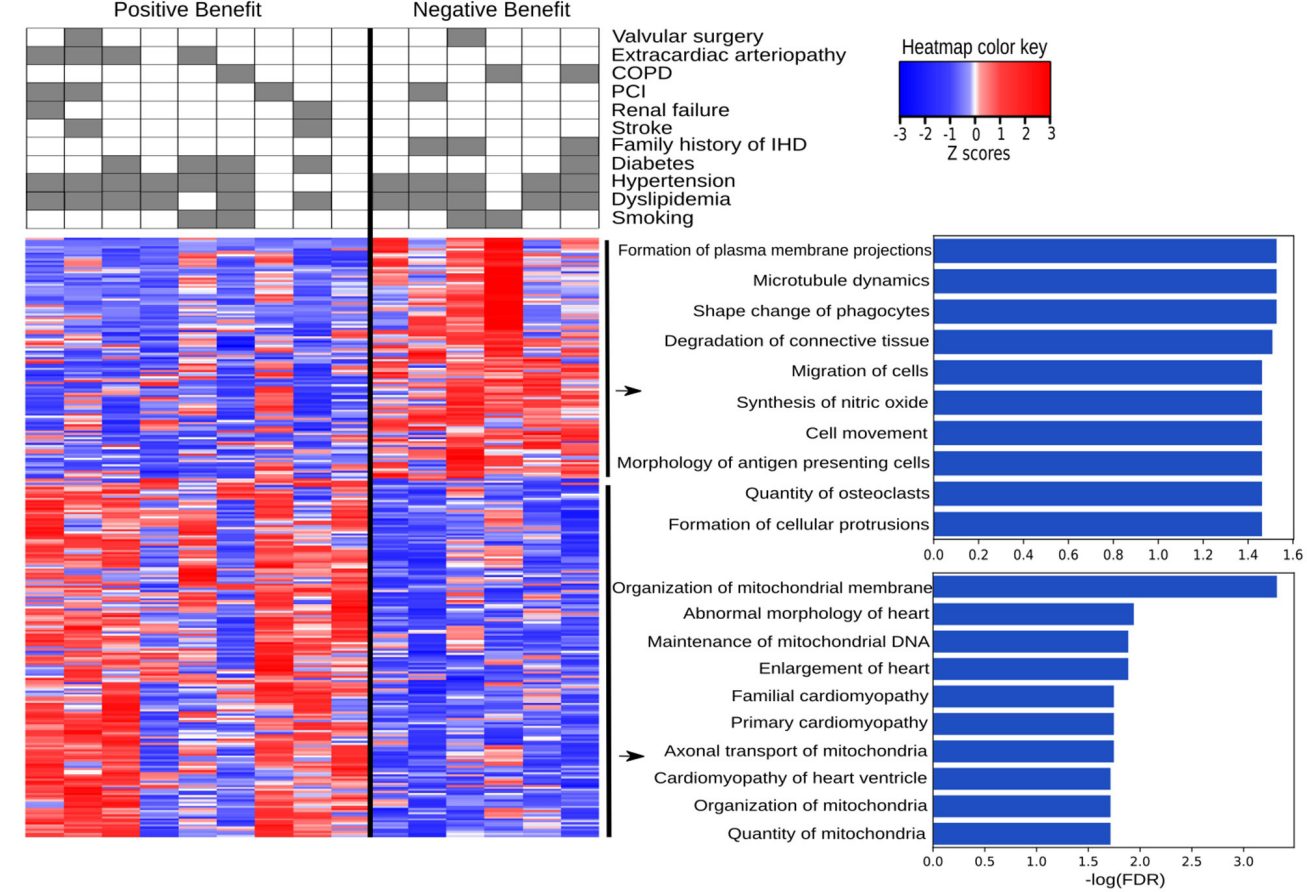
Pathways associated with a positive and negative benefit from surgery according to EF change. The heatmap provides a visual overview of the differences between two groups in terms of DEGs and clinically associated diseases, with color key representing the Z-score, and the top ten up and down regulated pathways are listed with their log(FDR) values.

**Figure 7:**
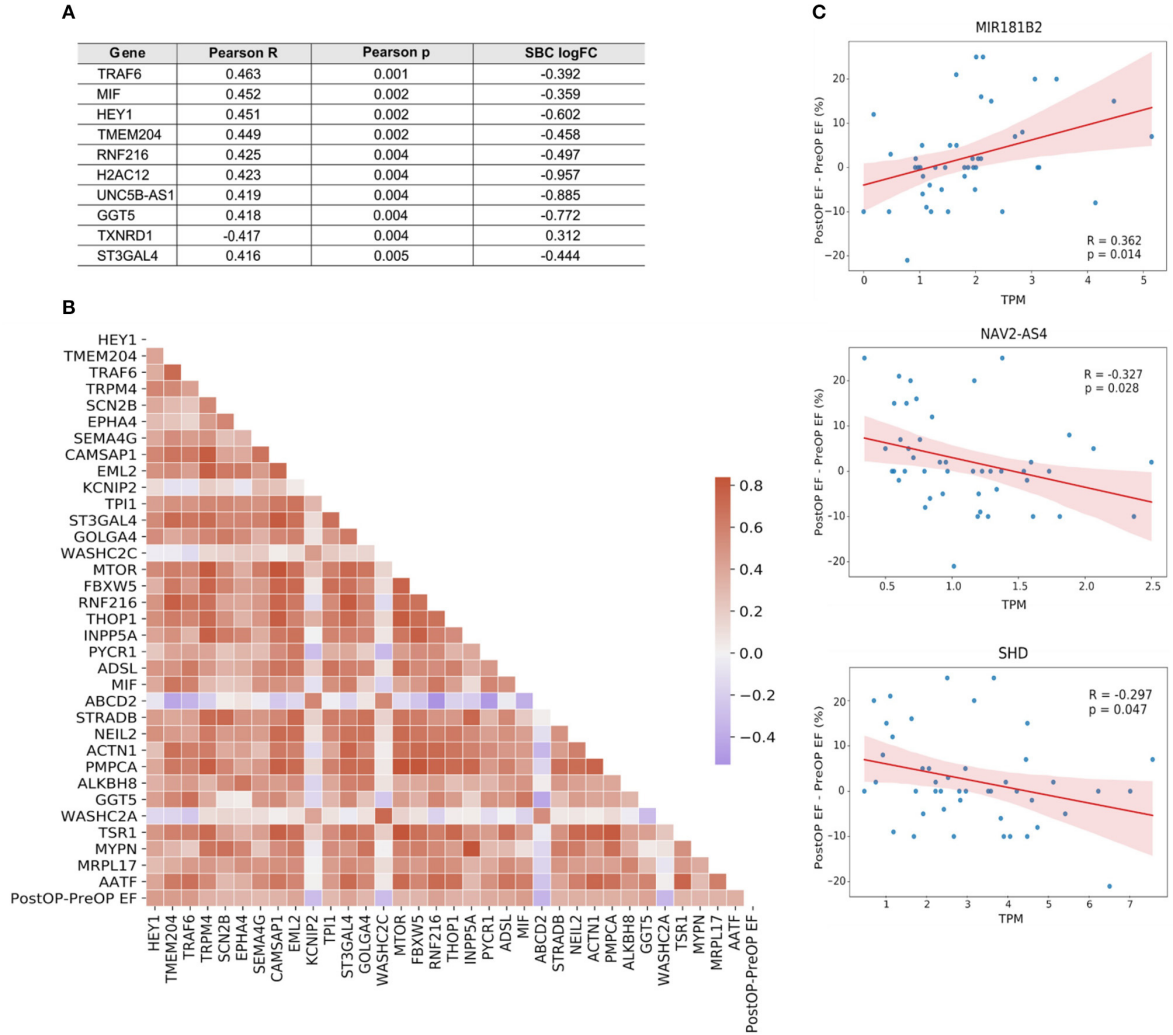
**(A)** Top 10 genes linearly correlated with the change in EF following surgery. **(B)** Correlation heatmap of the genes mapped to a GO biological process. Color key, correlation coefficient. **(C)** Correlation plots of the top three genes with the highest |logFC| between surgery benefit classes.

IHD patients were divided into groups based on a positive or negative benefit from surgery using the difference between pre- and post-operative EF values. Positive or negative benefit was assigned if the magnitude of the EF difference was 10% or more. Comparing the positive- and negative-benefit groups revealed a total of 271 DEGs. The clinical parameters among patients appear in the upper panel of Figure 6. Despite the clinical heterogeneity across patients, we identified 271 DEGs, among which 166 genes were downregulated and 105 genes were upregulated in the negative-benefit group compared to the positive-benefit group. The pathways associated with a surgical benefit (Figure 6) emerged from a functional enrichment analysis. The degradation of connective tissue (FDR = 0.031), cell migration (FDR = 0.034), and cell movement (FDR = 0.034) were upregulated in the negative-benefit group. The pathways related to an enlargement of the heart (FDR = 0.013), cardiomyopathy in the heart ventricle (FDR = 0.019), and the organization of mitochondria (FDR = 0.019) were upregulated in the positive-benefit group. Cardiac status-related demographics of benefit groups are presented in Supplementary Figure 4. Preoperative EF and first postoperative day CK-MBm were higher in the negative benefit group (Supplementary Figure 4A). No clinical observations of perioperative myocardial infarctions were documented. The magnitude of the EF change was significantly higher in the positive benefit group (Supplementary Figure 4). Linearly correlated genes with an EF change appear in Figure 7. The top three highest logFC genes comparing surgery benefit classes were mir-181b (Pearson *R* = 0.362, *p* = 0.014), *NAV2-AS4* (Pearson *R* = −0.327, *p* = 0.028), and *SHD* (Pearson *R* = −0.297, *p* = 0.047).

## Discussion

In this study, we present analysis of an IHD-selective RAA transcriptome and its relations to disease severity and outcome from cardiac surgery. In addition, identification of known IHD-associated pathways including changes in energy metabolism, cell structure, and surface proteins in the heart under pathological stress (16–21) further validated the applicability of the RAA as a surrogate source of information for enhancing our tissue-level understanding of human IHD.

We utilized the SYNTAX score, calculated based on coronary angiography, as a parameter for the complexity of coronary artery obstructions (22). Using ANOVA test, we detected significant results in gene expression between the SYNTAX scores and two IHD-associated DEGs. FUT10, or fucosyltransferase, catalyzes the transfer of a fucose residue (23), promotes adipocyte proliferation, and inhibits cell differentiation (24). Furthermore, it is a regulator of cell proliferation, particularly in stem cell self-renewal and in the maintenance of stem cell populations (25). In addition, FUTs are hallmarks of M1 inflammatory macrophages, and their expression, including that of FUT10, increases in inflammatory lesions (26). Recently, the post-translational role of FUTs in the glycosylation of immunoglobulins has garnered increasing interest. *FUT10* may serve as a possible target for therapeutic intervention (27). Interestingly, one protein-coding transcript (*FUT10-201*) incorporates a signal peptide, to potentially produce a secreted protein (28). *FUT10-201* transcript emerged as the most highly expressed in our sequencing analysis among sampled patients. This transcript and its coded (secreted) protein represent interesting candidates as tissue-level and/or circulating biomarkers for IHD. The expression of *FUT10* resulted significant with SYNTAX scores and correlated with preoperative NT-proBNP levels strengthening the correlation between increasing fucosylation and decreasing cardiac function in IHD.

*CSRNP3* gene expression resulted significant with the SYNTAX scores and correlated with preoperative CRP levels, indicating its potential to serve as an IHD-specific marker. *CSRNP3* is expressed in the nucleus, carries transcription factor activity (29), and links to apoptosis (30). Recently, *CSRNP3* was identified as one of eight overlapping genes between CD90pos MSCs (31) and the atherosclerosis associated gene expression dataset of aorta and fat tissue, STARNET (32). Our study further strengthens the association between *CSRNP3* and IHD/CAD, providing additional evidence of its cardiac tissue IHD-specific expression. Subanalysis correlating gene expression with proximal RCA occlusion strengthened the discovery of *HEY1*—a transcriptional suppressor and an atrial NOTCH, TGFbeta, and HIF1 pathway target gene—as an indicator of cardiovascular functional reserve in myocardial ischemia.

Upregulated mir-1254, mir-6769, and mir-4459 and their downstream target genes in IHD RAA showcase an interregulating network related to inflammation and immune regulation, cardiac hypertrophy, and adrenergic signaling.

The hsa-mir-1254 and its target gene *DBNL* regulate cell proliferation and immune responses (33–35), and increased expression of *DBNL* has been reported in experimental dilated cardiomyopathy (36). We found an IHD-associated downregulation of two other hsa-mir-1254 target genes, ALDOA (aldolase) and CLPP (caseinolytic mitochondrial matrix peptidase proteolytic subunit). In hypertrophic cardiomyopathy, reduced tissue expression of aldolase (37) and increased plasma concentrations of secreted aldolase in cardiomyocyte-derived exosomes have been reported (38, 39). In our data, reduced *ALDOA* expression in RAA was associated with IHD, suggesting a similar tissue-level regulation to that observed in hypertrophic myocardium, possibly indicating a common metabolic dysregulation in these diseases. Reduced expression of *CLPP*, as found here to be IHD-associated, has also been reported in knockout mice to decrease formation of cardiac mitochondrial respiratory supercomplexes, T-cell activation, and increased production of mediators of inflammation and immunity contributing to inflammatory tissue injury (40, 41). Furthermore, reduced cardiac CLPP expression may contribute to mitochondrial dysfunction and tissue injury in IHD, but its actions in the heart seem to depend on the disease background and require further elucidation (42). The RAA IHD-associated miRNA hsa-mir-6769a regulates the expression of synaptotagmin-7 (*SYN7*). *Syn7* knock-out mice present with increased cardiac inflammation and deposition of collagen (43) suggesting that increased hsa-mir-6769a may drive myocardial fibrosis, thus contributing to fibrosis in RAA. Another IHD-associated RAA miRNA found in our study, hsa-mir-4459, has been reported to suppress inflammatory reactivity and autophagy of vascular endothelial cells through inhibition of its proinflammatory target gene *LARP-1* (44) thereby possibly also contributing to regulation of the inflammatory tissue response in RAA under IHD.

We found IHD-associated reduced expression of the insulin-like growth factor-1 receptor (IGR-1R) signaling effector (45) hsa-mir-6815 in the RAA samples. Interestingly, it has been shown that macrophages deficient of IGR-1R accelerate atherosclerosis and increase atherosclerotic plaque vulnerability (46) and that the hsa-mir-6815 response is activated under hypoxia and coagulation (47). Taken together with our results, these findings suggest that these RAA miRNAs can contribute to the tissue level pathological process observed in IHD.

Our results suggest that IHD is associated with an increased expression of transcriptional repressors, implying a complex modulation of metabolic inflammation or macrophage polarization through transcription factor activity and putative aggravation through suppressing inflammatory repressors (48). For example, *KLF10* and *KLF11* are tightly linked to vascular inflammation, but also carry an anti-inflammatory role in experimental disease models and in *in vitro* studies (49). Our results associated *KLF10* and *KLF11* with IHD, thereby indicating a further complexity to the transcriptional regulator networks in the multimodal pathology of IHD. This agrees with our findings of IHD-associated increases in the expression of the inflammation-associated transcriptional activator *RUNX1* (50) together with the transcription regulator *MYEF2* shown to modify *RUNX1* function and target gene selectivity (51). Thus, it seems feasible to assume a more elaborate and environmentally sensitive mechanism for these factors in complex metabolic diseases such as IHD (52).

We then analyzed the RAA DEGs that were associated with the patients' functional cardiac benefit from surgery. Despite the complexity of categorical EF use, we were able to discover DEGs that were related to absolute EF change and correlated with surgery benefit without EF categorization. One such DEG, *SHD*, encodes for an SH2 homology domain-containing protein SHD, which interacts with key signaling phosphoprotein receptors such as c-MET (53). Also the cis-natural antisense transcript *NAV2-AS4* significantly correlated with a benefit from surgery. *NAV2* expresses in the heart (54), but its role has remained unspecified. It has, however, been shown to regulate cell adhesion and neurite outgrowth (55). Hsa-mir-181 expression levels significantly negatively correlated with cardiac surgery benefit. Changes in mir-181 expression have been associated with the early stages of heart failure (56), regulating thrombin-induced endothelial cell activation and dysfunction, arterial thrombosis pathology, and promoting atherosclerosis (57). Moreover, diminished mir-181b levels have correlated with the ability to remodel the extracellular matrix affecting vascular stiffness via TGF-β signaling (58), while circulating mir-181b levels have been associated with diabetic cardiomyopathy (59). Due to mir-181's ability to reflect cardiac status, its expression represents a potential novel marker for predicting cardiac surgery benefit. As associated with a functional benefit from surgery, the expression of these DEGs can serve as an indicator for the patient's cardiac functional recuperation reserve.

This study provides a rationale for the utilization of RAA tissue RNA profiling in the search of potential IHD biomarkers. We meticulously collected available clinical, imaging, and laboratory data from electronic healthcare records as well as documented patient drug treatments at a high resolution. All analyses progressed according to clinical guidelines for patient treatment and, thus, any study-guided analysis bias can be eliminated. The classification of medications according to their ATC class and the standardization of doses using DDD conversions relying on the InnoLIMS Medical software platform enabled us to compare drug treatments at various time points and intervals. We also performed an initial immunohistochemical validation of results for early preliminary confirmation of our results.

Our study also has several limitations. The total number of patients in the study and the relatively low number of non-IHD patients limit our analytical power. Another limitation lies in the retrospective collection of clinical data from healthcare databases, possibly leading to incomplete coverage and interobserver variability in the clinical measurements. Lastly, immunohistochemical validations were performed on a limited number of patient samples, with varying levels of IHD according to the SYNTAX scores. Non-IHD patient samples or samples from donors without cardiac disease were not used. Thus, validation on a larger cohort is necessary. Our recently initiated IHD-EPITRAN project (ClinicalTrials.gov identifier NCT04533282) will address this limitation (60).

Based on their differential expression in the RAA tissue of patients with or without IHD, we present here a specific set of IHD-associated RNAs expressing significantly differently when compared to coronary artery disease complexity, and correlating with CRP, NT-proBNP, and surgery outcomes. Specifically, *FUT10* and *CSRNP3* represent lucrative candidates as IHD biomarkers.

## Data Availability Statement

The RNA sequencing data is available from the Gene Expression Omnibus database repository under the identification GSE173594, https://www.ncbi.nlm.nih.gov/geo/query/acc.cgi?acc=GSE173594.

## Ethics Statement

The studies involving human participants were reviewed and approved by the Operative Ethics Committee of the Hospital District of Helsinki and Uusimaa. The patients/participants provided their written informed consent to participate in this study.

## Author Contributions

MKu, AV, AH, EK, and JL: conceptualization. MKa, AH, EK, AV, ML, AN, and JL: methodology. SM, AN, KT, ML, TN, JL, TO, MKa, AE, and NT: investigation. SM, EK, NT, and AE: visualization and writing—original draft. EK, AV, AH, MKu, and NT: funding acquisition and project administration. EK, AV, AH, and NT: supervision. SM, AE, EK, NT, ML, TN, KT, TO, MKa, AV, JL, MKu, AH, and NT: writing—review and editing. All authors have reviewed and approved the manuscript.

## Funding

This study was funded by the Finnish Medical Foundation (SM), the Aarne Koskelo Foundation (SM), the Finnish Foundation for Cardiovascular Research (ML), Finnish government subsidies for medical research block grants (EVO, AH TYH2014207 and MK TYH2015311), the Finnish Funding Agency for Technology and Innovation (EK 3137/31/2013), UNESCO-L'Oréal National Fellowship for Women in Science (NT), UNESCO-L'Oréal International Rising Talent Fellowship (NT), and TUBA-GEBIP (NT).

## Conflict of Interest

The authors declare that the research was conducted in the absence of any commercial or financial relationships that could be construed as a potential conflict of interest.

## Publisher's Note

All claims expressed in this article are solely those of the authors and do not necessarily represent those of their affiliated organizations, or those of the publisher, the editors and the reviewers. Any product that may be evaluated in this article, or claim that may be made by its manufacturer, is not guaranteed or endorsed by the publisher.
