# Supplementary material for: Ischemic Heart Disease Selectively Modifies the Right Atrial Appendage Transcriptome
Source: Front Cardiovasc Med. 2021 Dec 2;8:728198. doi: 10.3389/fcvm.2021.728198 (PMC8674465; doi:10.3389/fcvm.2021.728198)
Supplement: Supplementary file 1 [file Data_Sheet_1.DOCX]

**Mulari et al**

**Online Supplement**

**Supplementary Table 1.** Comprehensive patient characteristics. Statistical differences in continuous variables calculated using the Kruskal–Wallis test, while the statistical differences in the counts were calculated using the Fisher's exact test. Continuous variables expressed as mean ± SD and range (min–max), while counts are expressed as the number of subjects (percentage of total cohort). *p < 0.01.


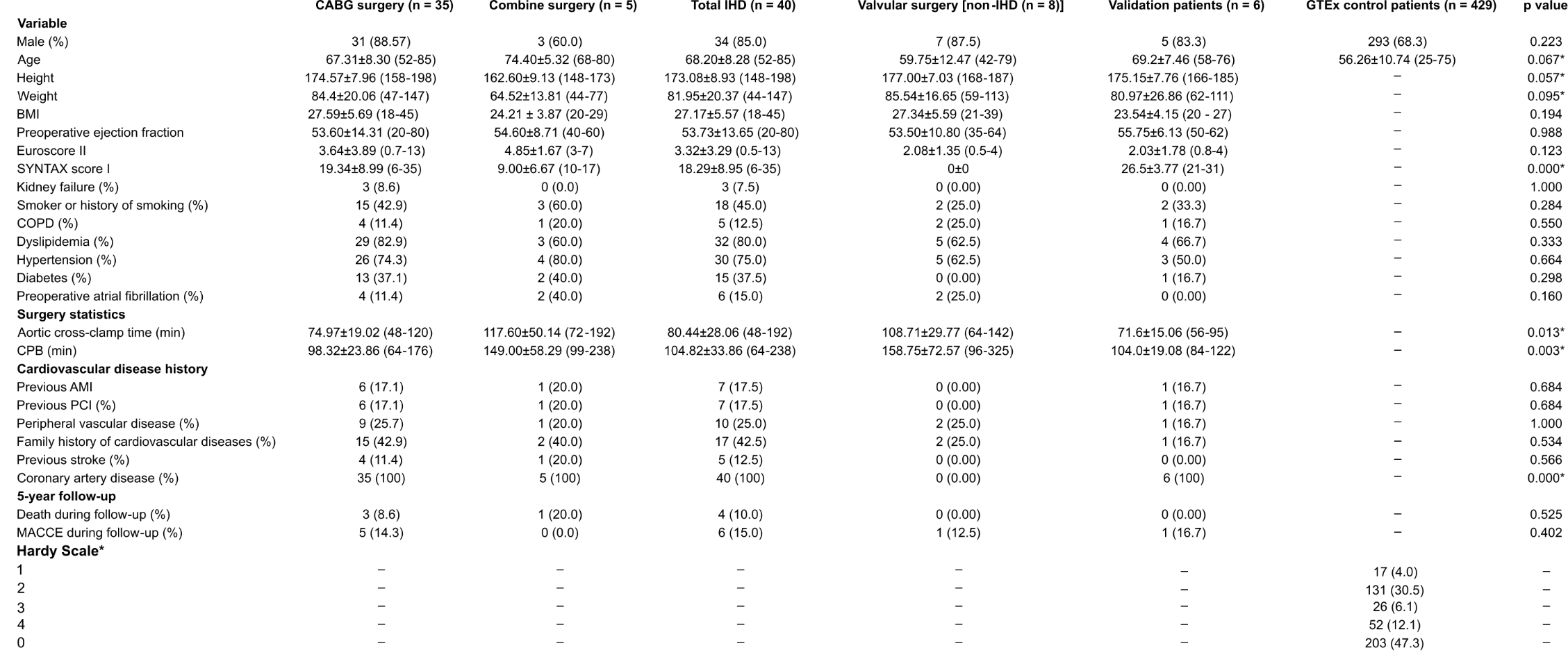


*Cases with cardiac background were excluded. Hardy scale: 1 = violent and sudden death due to blunt trauma, suicide, and accident; 2 = sudden death from natural causes, such as cerebrovascular events; 3 = intermediate death (patients who were ill, but death was unexpected); 4 = slow death after long illnesses, such as cancer; 0 = ventilator case, all cases that were on a ventilator immediately before death. Abbreviations: AMI, acute myocardial infarction; BMI, body mass index; COPD, chronic obstructive pulmonary disease; CPB, cardiopulmonary bypass; MACCE, major adverse cardiovascular and cerebrovascular event; PCI, percutaneous coronary intervention.


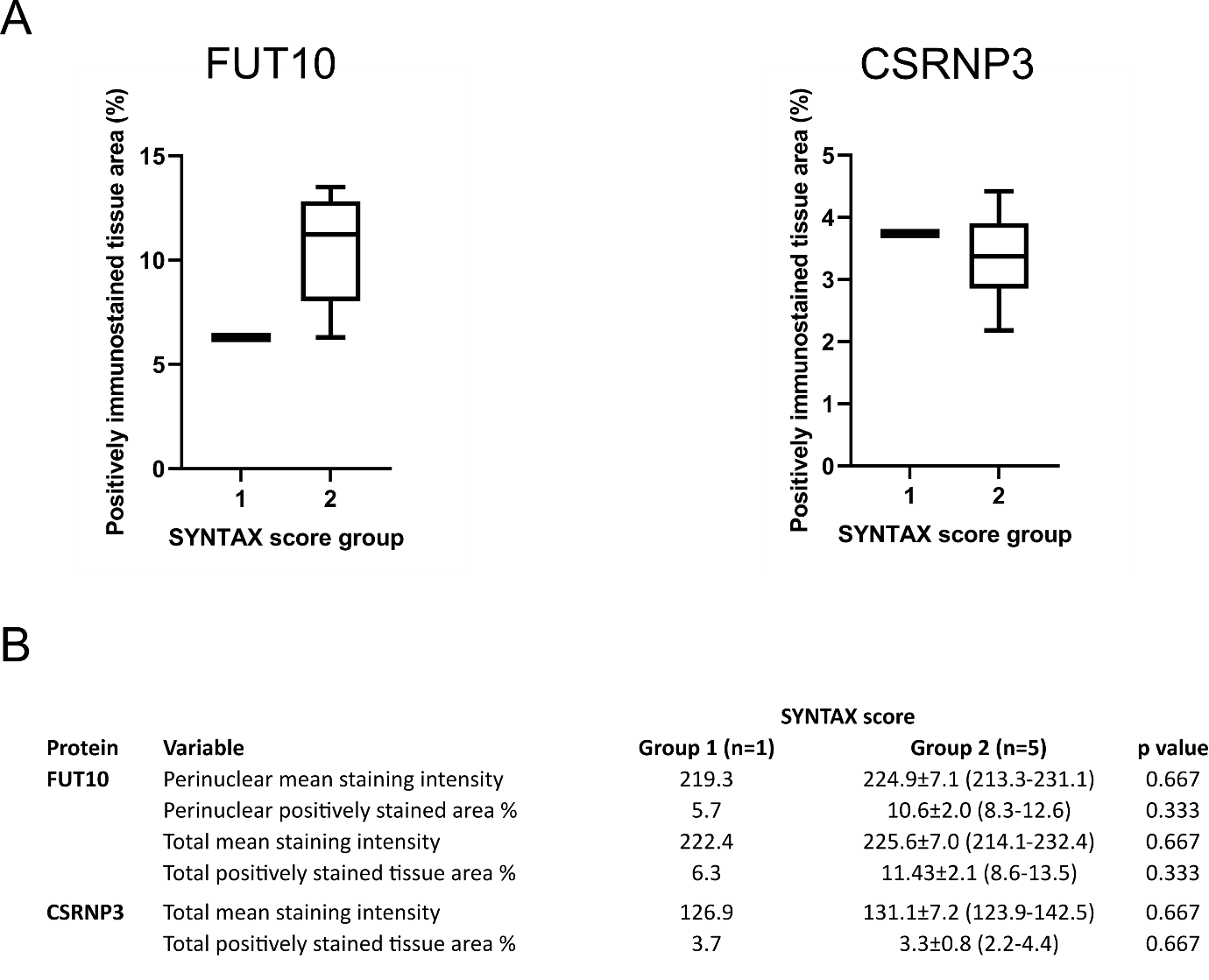


**Supplemental Figure 1. A. Box plots illustrate the expressions of FUT10 and CSRNP3 in the patient validation samples**, reported as percentage of the total sample area stained. **B. Results of IHC analysis.** SYNTAX score group 1 represents SYNTAX score values ≤ 22, while SYNTAX score group 2 represents SYNTAX score values 23 – 32. Statistical differences were calculated using the Mann–Whitney U-test.


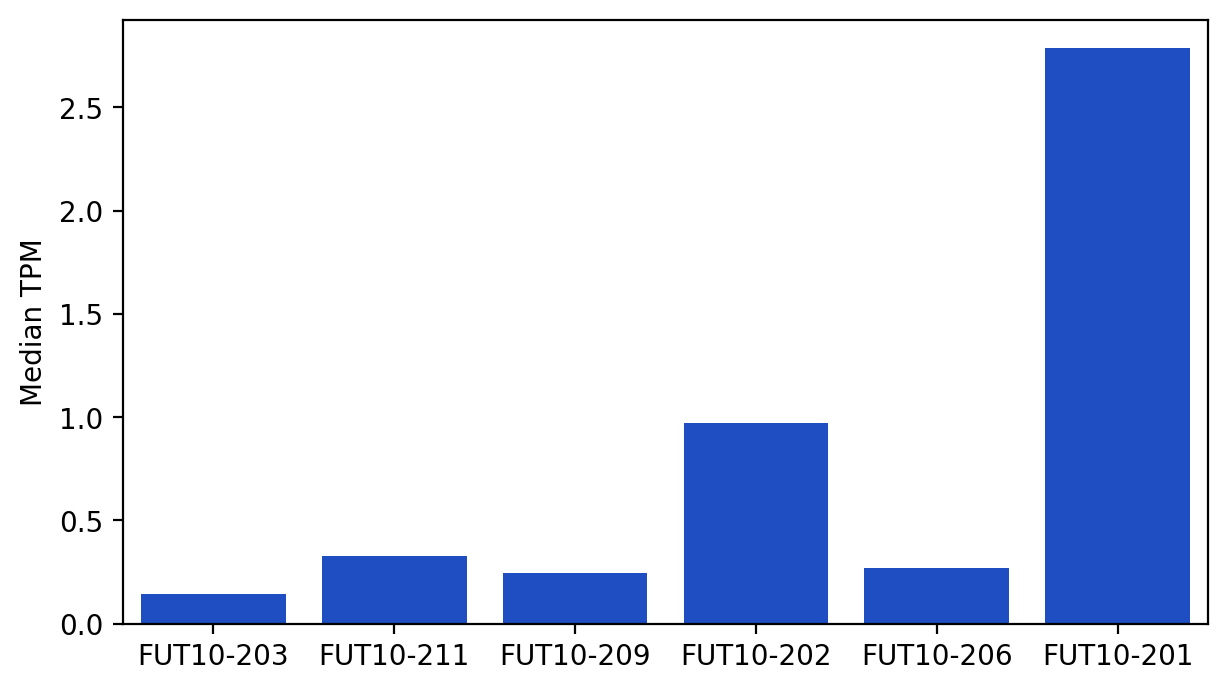


**Supplemental Figure 2.** The median expression levels for all of the expressed transcripts (TPM > 0.1) of *FUT10* among study patients**.**


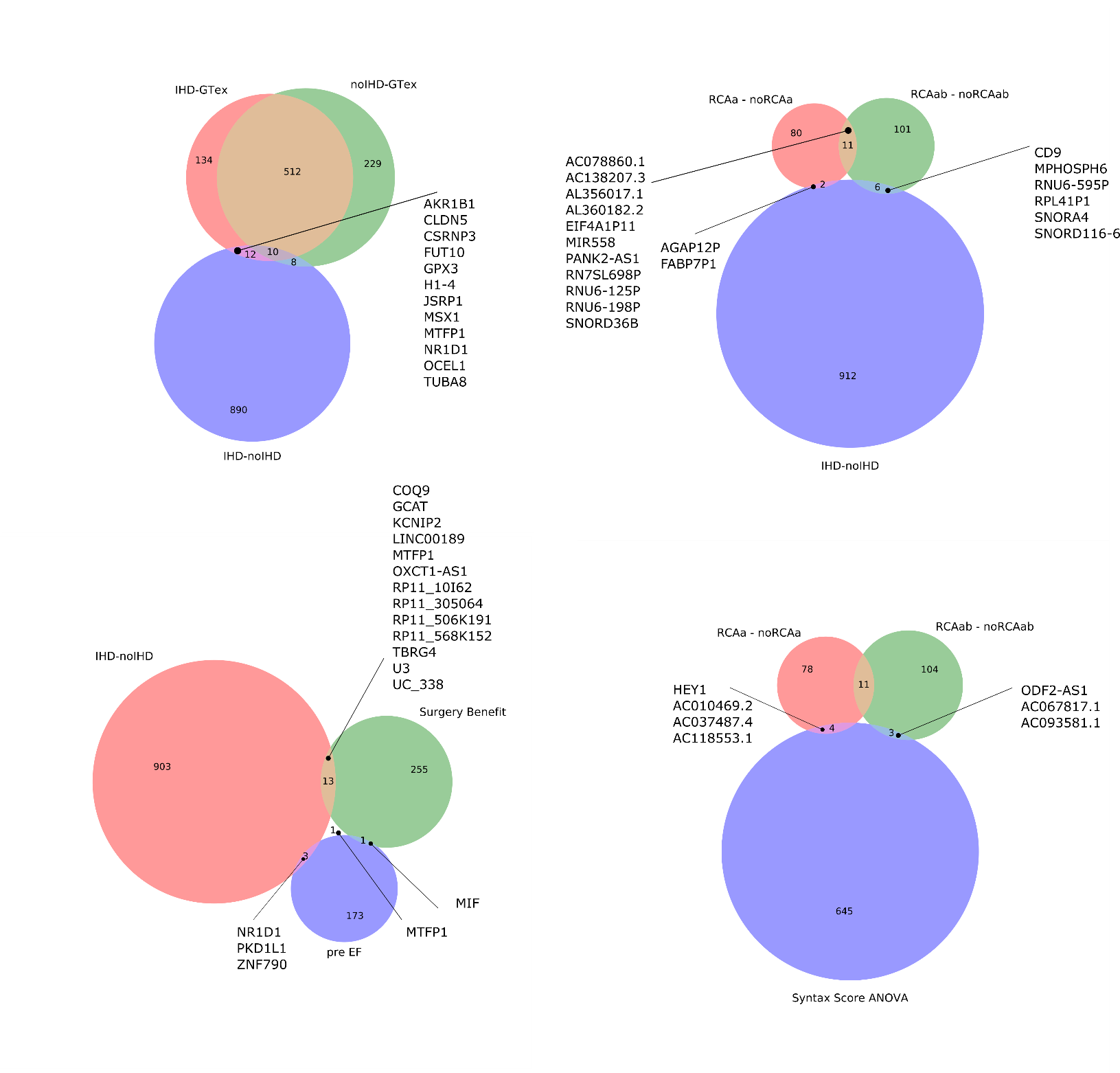


**Supplemental Figure 3.** **Common genes that were observed between the RCA analyses.** As an additional analysis, differentially expressed genes between RCAa occlusion and no RCA occlusion & RCA a or b occlusion and no RCA a or b occlusion were found using permutation test ( FDR < 0.05, |logFC| > 0.5). Common genes were reported between the analyses. Abbreviations: RCA, right coronary artery.

**
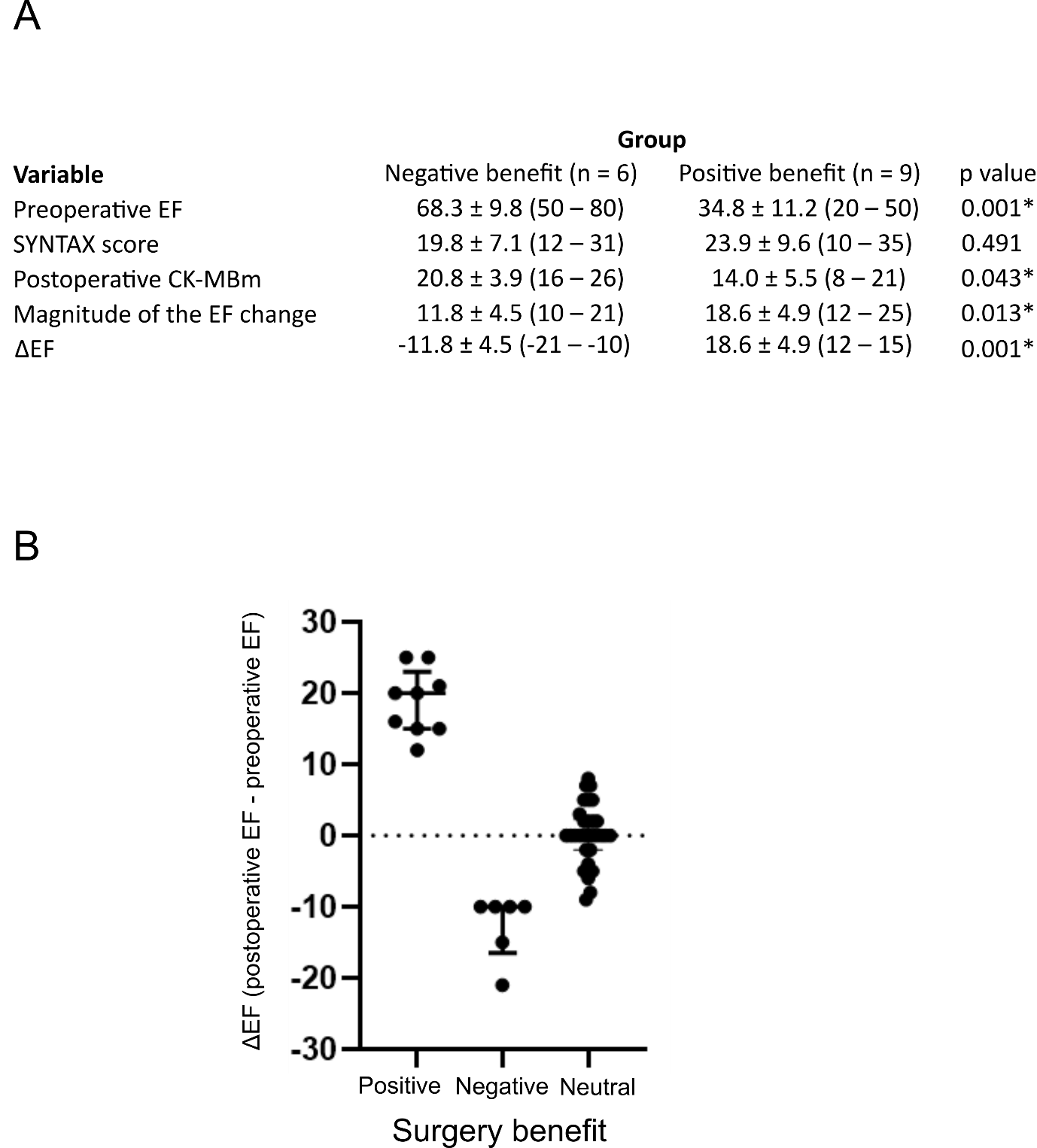
**

**Supplemental Figure 4. A. Benefit group demographics.** CK-MBm levels were not diagnostic for perioperative myocardial infarction (11,12). Abbreviations: EF, ejection fraction; CK-MBm, creatine kinase myocardial band. **B. Scatterplot showing the change of EF in benefit groups.**
